# Periodicity Pitch Perception Part III: Sensibility and Pachinko Volatility

**DOI:** 10.3389/fnins.2022.736642

**Published:** 2022-03-08

**Authors:** Frank Feldhoff, Hannes Toepfer, Tamas Harczos, Frank Klefenz

**Affiliations:** ^1^Advanced Electromagnetics Group, Technische Universität Ilmenau, Ilmenau, Germany; ^2^Fraunhofer-Institut für Digitale Medientechnologie, Ilmenau, Germany; ^3^Auditory Neuroscience and Optogenetics Laboratory, German Primate Center, Göttingen, Germany; ^4^audifon GmbH & Co. KG, Kölleda, Germany

**Keywords:** Pachinko volatilities, sensibility, stateful temporal logic, dendritic back-propagation computation, inter spike intervals

## Abstract

Neuromorphic computer models are used to explain sensory perceptions. Auditory models generate cochleagrams, which resemble the spike distributions in the auditory nerve. Neuron ensembles along the auditory pathway transform sensory inputs step by step and at the end pitch is represented in auditory categorical spaces. In two previous articles in the series on periodicity pitch perception an extended auditory model had been successfully used for explaining periodicity pitch proved for various musical instrument generated tones and sung vowels. In this third part in the series the focus is on octopus cells as they are central sensitivity elements in auditory cognition processes. A powerful numerical model had been devised, in which auditory nerve fibers (ANFs) spike events are the inputs, triggering the impulse responses of the octopus cells. Efficient algorithms are developed and demonstrated to explain the behavior of octopus cells with a focus on a simple event-based hardware implementation of a layer of octopus neurons. The main finding is, that an octopus' cell model in a local receptive field fine-tunes to a specific trajectory by a spike-timing-dependent plasticity (STDP) learning rule with synaptic pre-activation and the dendritic back-propagating signal as post condition. Successful learning explains away the teacher and there is thus no need for a temporally precise control of plasticity that distinguishes between learning and retrieval phases. Pitch learning is cascaded: At first octopus cells respond individually by self-adjustment to specific trajectories in their local receptive fields, then unions of octopus cells are collectively learned for pitch discrimination. Pitch estimation by inter-spike intervals is shown exemplary using two input scenarios: a simple sinus tone and a sung vowel. The model evaluation indicates an improvement in pitch estimation on a fixed time-scale.

## 1. Introduction

**Figure 1:**
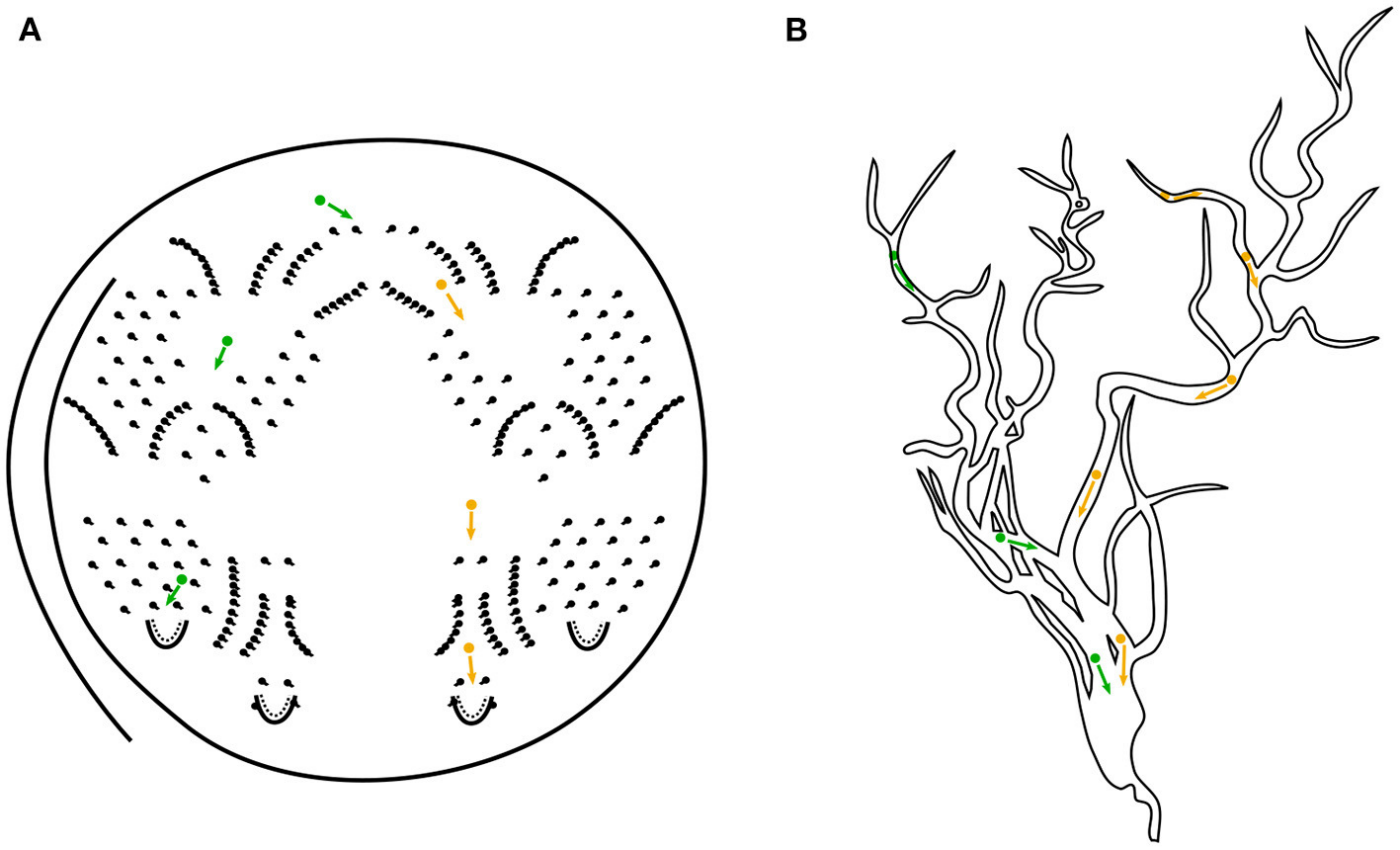
**(A)** Schematical illustration of the Pachinko game. A ball is shot *via* a metal track to the top of the board and it finds its way through several patterns of small obstacle pins back to the release hole by gravitational force. This aligns well with the pathfinding mechanism of our octopus' model letting aside the proposed new learning mechanism. Colors green and orange indicate two possible ways a ball could choose. For comparison, in **(B)** the vesicle transport in a dendritic tree toward the octopus' cell's soma is depicted.

Octopus cells are tonotopically arranged in the cochlear nucleus and connected to several auditory nerve fibers *via* their dendritic trees. Phenomenologically, octopus cells fire in the presence of broadband acoustic stimuli in response to constellations of spike trains from the associated auditory nerve fibers in their local receptive fields. The hypothesis of this work is that an octopus' cell responds to broadband stimuli by following a specific hyperbolically shaped trajectory that is observable in the cochleagrams. Due to the fact, that octopus neurons play a key role in several parts of acoustic cognition of sounds and speech they are candidates for a deeper investigation toward fast and energy-efficient computing systems. In previous articles in the series of periodicity pitch perception (Harczos and Klefenz, 2018; Klefenz and Harczos, 2020) it is shown, that octopus neurons in a network topology can process the acoustic signals efficiently and detect pitches with an astonishing accuracy. In this work a discrete, event-based approach is presented that has its main focus on a simplistic model which can be easily implemented in hardware. The main idea is to enforce synchronous events by retarding signals resulting from the cochlea traveling wave delays. The delay trajectories are bent straight in time due to differences in the local distance between the corresponding ANF and the soma of the octopus' cell. This results in isochronous arrival times at the soma and triggers a depolarization event of the soma's membrane. The underlying simulation algorithm of spatio-temporal template matching is explained in section 3 in detail. One guiding question is: How does an octopus' cell becomes selective for a specific trajectory? We will give an answer to the question, whether the octopus' cell is able to improve the pitch detection significantly by using only locally available information. We show that an octopus' cell is able to learn the trajectory by a new postulated hardware friendly spike-timing-dependent plasticity (STDP) learning rule (Gerasimova et al., 2021). The synaptic connection on the dendritic tree projecting to ANF inputs are strengthened, when the criterion of isochronicity at the soma is met. This is achieved by using a spiking-neuron model with a leaky-integrating soma, a connectome varying in length with an inherent backpropagation procedure. An action potential is triggered when the cumulative potentials at the soma are above the depolarization threshold (Lubejko et al., 2019). The soma detects coincidences depending on dendritic tree morphology and dendritic ion channel flux velocities, with pre-synaptic early arrivals being compensated by forward positions of synaptic connections along the dendritic tree (Leão, 2019; Radler et al., 2020). Its synchrony transfer function will be investigated in detail by which the octopus' cell comprehends a coherent constellation, in which associated active inputs form a feature. The octopus' cell's soma is a gate with an ultra-precise switch point (Lu et al., 2018; Lubejko et al., 2019). The gate switches at an unique moment, when the non-static balance of excitatory and inhibitory vesicles is broken by any spillover vesicle. Due to the analogy of this process to a game called Pachinko, we gave this procedure the name Pachinko volatility (see Figure 1).

## 2. Biologically Motivated Background

To underpin our approach, we refer to some basic findings that explain its plausibility and feasibility at the neurophysiological level. The connectome of neuronal ensembles is orchestrated by neurite guidance, axonal and dendritic branching, synaptogenesis, and synaptic plasticity (Mrsic-Flogel and Bonhoeffer, 2012; Rajani et al., 2020; Rubio, 2020). The assembly of specific neuronal circuits depends on the expression of complementary molecular programs in presynaptic and postsynaptic neurons (Keable et al., 2020). Proteins are synthesized locally in different subcellular compartments such as dendritic shafts and spines, triggered by molecular signals such as neurotrophins, brain-derived neurotrophic factor, metabotropic glutamate receptor agonists, or by electrical stimulation (Ribeiro et al., 2019; Wu et al., 2019). Neuronal activity regulates the selection of unique initiation sites and up- or down-regulates the protein translation machinery in nascent synapses (Munz et al., 2014; Eberhardt et al., 2019). Auditory stimulation regulates synaptic development by triggering local signaling events. Unique activity patterns and signaling pathways fine-tune synapses (Winnubst et al., 2015; Sakai, 2020; Scholl et al., 2021).

### 2.1. Time-Based Auditory Modeling

High-fidelity audio recording and playback technologies use a minimum standard sampling rate of 44.1 kHz. How auditory neuron ensembles cope with this sampling rate was not clear until recently, when a computational neuroscience model illustrated periodic pitch perception on a microsecond scale (Klefenz and Harczos, 2020). In the model, equilibrium tipping points are evoked by an excess of excitatory vesicles relative to the currently available reservoir of inhibitory vesicles at the soma of inferior colliculus (IC) neurons and recorded with extreme temporal precision. Oscillations of octopus neurons are perceived by IC neurons as differentiable pitch sensations. Sounds are transformed into spike-based event representations by a bio-plausible, neuro-physiologically parameterized auditory model (Harczos, 2015; James et al., 2017; Cramer et al., 2020; Gutkin, 2020; Baby et al., 2021; Gutierrez-Galan et al., 2021; Saddler et al., 2021). First, the auditory model computes spike train patterns for auditory nerve fibers (ANFs). The auditory nerve divides into several sub-nuclei of the cochlear nucleus. In the dorsal cochlear nucleus, octopus cells receive ANF spike trains in their tonotopically arranged local receptive fields (Kane, 1973; Spencer et al., 2018). The model calculates the periodicity of pitch from the rhythmic oscillations of the octopus cells. The inter-spike intervals (ISI) of octopus cells are measured by batteries of interval-tuned neurons (ITNs) by encoding the interval durations as first spike latencies (FSLs) (Aubie et al., 2009, 2012). Aubie's model is formulated in NEURON with excitatory NMDAR/AMPAR GABAergic inhibition^1^ (Kopp-Scheinpflug et al., 2018) and has been adapted and optimized to work reliably in the microsecond range (Klefenz and Harczos, 2020). For better understanding, some parts of the two articles in the Periodicity Pitch Perception series (Harczos and Klefenz, 2018; Klefenz and Harczos, 2020) are recapitulated and some of the figures are reprinted.

### 2.2. Synaptic Plasticity

Synaptic plasticity depends on its dendritic location, the detailed timing protocol of pre- and postsynaptic events, and the temporal states of postsynaptic hyper/depolarizations (Bach and Kandler, 2020). STDP signaling cascades enlarge dendritic spines through polysome association (Wierda et al., 2020). Initially, the synapse is preactivated by the release of neurotransmitters into the synaptic cleft when triggered by an ANF spike and neurotransmitters open (NMDA) channels for Ca_2+_ influx. Subsequently, STDP requires postactivation, which occurs through the generation of a somatic sodium (Na_+_) spike that propagates back into the dendrites (Winters and Golding, 2018). The briefly previously presensitized synapse is potentiated by the passing backpropagating spike, as these concurrent pre/post stimuli trigger the influx of calcium into the spike head of the synapse (Franzen et al., 2020; Kladisios et al., 2020). If it is an inhibitory synapse, it will be suppressed. If a group of synapses is activated by presynaptic events but does not elicit a somatic spike, its weighting is lowered because the postcondition for potentiation is absent. The membrane voltage at the synapse can be modeled as an algebraic equation based on the summation of an excitatory postsynaptic potential (EPSP) and a backward propagating action potential (bAP) (Jahr and Stevens, 1993; Griffith et al., 2016). The stimuli are membrane depolarization due to an EPSP and a bAP about 2 ms apart. When the EPSP arrives 2 ms before the bAP, the maximum possible membrane depolarization is elicited (Hu and Bean, 2018). In general, a single general plasticity rule is sufficient to reproduce different results of plasticity experiments at different dendritic sites, allowing unification of classical STDP- and Ca_2+_-based rules. The plasticity rule can be easily combined with detailed neuron models to study both STDP and plasticity mediated by dendritic Ca_2+_ and Na_+_ spikes, NMDA spikes, and synaptic cluster activation (Palmer et al., 2014; Foncelle et al., 2018; Augusto and Gambino, 2019). When a pre-post spike pair is insufficient to trigger potentiation, spike triplets are. The solution approach combines dendritic back-propagation with triplet spike timing dependent plasticity signaling. Potentiation is, therefore, possible when isolated spike triplets are present (pre-post-post or post-pre-post). A pre-post-post protocol triggers much more post-LTP than a post-pre-post protocol. Synaptic potentiation is triggered by spike triplets consisting of one presynaptic and two postsynaptic spikes (Pfister and Gerstner, 2006). Synaptic plasticity is discussed with special emphasis on the role of NMDAR and AMPAR signaling cascades (Rajani et al., 2020). The relative position of postsynaptic AMPAR domains with respect to presynaptic release sites and the molecular basis of such co-organization have been investigated in several studies (Goncalves et al., 2020). Bell et al. (2019) model spiny heads along dendrites by boundary conditions at the plasma membrane (PM) and spiny apparatus (SpApp) in a spatial multicompartment reaction-diffusion model of calcium dynamics in three dimensions with different flux sources, including N-methyl-D-aspartate receptors (NMDARs), voltage-sensitive calcium channels (VSCCs), and various ion pumps at the plasma membrane (PM) (Ingólfsson et al., 2017; Cheng and Smith, 2019; Ohadi et al., 2019). AMPA-type glutamate receptors (AMPARs) mediate fast excitatory synaptic transmission (Choquet, 2018). AMPARs are concentrated within the postsynaptic density (PSD) in small nanoclusters of approximately 80 nm in size, containing an average of 20 receptors (Masugi-Tokita et al., 2007; Fukata et al., 2013; MacGillavry et al., 2013; Nair et al., 2013). Because of AMPAR's low glutamate affinity glutamate must be released precisely in front of AMPAR nanoclusters and, therefore, the relative positioning of pre-synaptic AMPAR‘s release sites with respect to AMPAR nanoclusters is the critical factor for synaptic transmission (Choquet and Hosy, 2020). Active glutamate release sites are co-localized with the presynaptic active zone protein RIM and aligned with AMPAR nanoclusters (Beique et al., 2006). To model the effects of delocalization of AMPAR nanoclusters from presynaptic glutamate release sites, Haas et al. (2018) performed Monte Carlo-based simulations using the MCell/CellBlender simulation environment (http://mcell.org) with MCell version 3.3, CellBlender version 1.1, and Blender version 2.77a (http://blender.org). Kinney et al. (2013) and Bartol et al. (2015b) obtained synaptic shape and peri-synaptic environment from 3D electron microscopy images (Kinney et al., 2013; Bartol et al., 2015a,b). Jonas et al. (1993) determined the chemical kinetic properties of AMPAR using an established model (Jonas et al., 1993), and Nair et al. fitted the kinetic parameters to both the recorded mEPSCs and the AMPAR organization map obtained from d-STORM data (Nair et al., 2013). In their simulations, Savtchenko et al. set the number of glutamate molecules released to 1,500, 2,000, 3,000, or 4,500 to be within the range of the estimated amount per presynaptic vesicle (Savtchenko et al., 2013). The simulations calculated the number of open AMPARs when vesicles containing the different amounts of glutamate were released upstream of a single AMPAR cluster or up to 200 nm away from the cluster center, varied with a step size of 50 nm. Nair et al. (2013) adjusted the AMPAR rate constants in their model using simplex optimization with minimal least squares to best fit the shape of the AMPAR current. Jonas et al. (1993) set the initial parameter values with the release of glutamate directly across the cluster, using n_Glu_ = 3,000, n_AMPAR_ = 25 in the cluster. The AMPAR activation time courses of 100 simulation trials were averaged at each release site. Computer modeling predicts that a lateral shift of approximately 100 nm between AMPAR nanoclusters and glutamate release sites results in a significant reduction in AMPAR-mediated currents (best fit parameter values in Kim et al. (2018). In the method part the functional octopus' model will be unrolled. Clues and pointers for hardware realizations of the octopus' cell are stated in the discussion section.

### 2.3. Distributed Signal Transduction in Dendritic Trees

The function of the octopus' cell is to permanently observe spatiotemporal trajectories in its local receptive field and to selectively respond to a trajectory with a specific hyperbolic shape. Constellations of distributed, cascaded synaptic input activations predetermined by the hyperbolic shape of a trajectory can lead to coincidence detection at the soma by triggering a spike for that event. The coincidence detection function of the octopus' cell is based on the morphology of its dendritic tree, the distributions of synaptic inputs along the dendritic trees, the event-based cascades of synaptic input activations and the local signal propagation velocities in the dendritic branches (Remme et al., 2018). ANFs innervate octopus' cells through synaptic connections along their dendritic trees. At the synaptic sites, activation triggers dendritic spikes that propagate to the soma. In this way, even the most distant synapses influence the electrical potential at the soma. In the simplest case, a single distant synaptic input is attenuated and low-pass filtered before it reaches the soma. A single EPSP remains below threshold, and most of the collective synaptic potentials are too weak on their own to trigger a somatic action potential, but some constellations have converging EPSPs that sum at the soma and trigger an AP (Kladisios et al., 2020). The dendritic calculation evaluates the threshold crossing condition as a function of the actual sum of EPSPs arriving simultaneously at the soma. The potential flow calculation can be performed for each local dendrite segment by assigning a cable conductance value. These conductance values determine the local signal propagation velocities. The conductances allow the computation of coincidences of temporally consecutive synaptic inputs within a branch, between different branches, or throughout the dendritic tree (Li et al., 2019). With precisely activated inputs cascaded in time, the potential currents in the dendritic branches converge according to Kirchhoff's current law and swell until they overflow at the soma. An Na_+_ or an NMDA (N-methyl-D-aspartate) or a Ca_2+_ ion channel could be one of the transmissive cable lines (Spruston et al., 1995a).

### 2.4. Modeling Post-signaling in STDP by Back-Propagation Potentials in the Dendritic Tree

ANF spike trains trigger dendritic spikes at synaptic sites distributed along the dendrite tree. The collaboration of synaptic inputs from multiple dispersed sites is required for somatic spiking (Urbanczik and Senn, 2014). Some spatio-temporal coherent constellations of dendritic spikes arrive synchronously at the soma and reliably generate temporally precise APs and thus event-based timestamps. Complementarily, APs fired at the axon hillock bounce back in the opposite direction, back-propagate along the dendrites and cause postsynaptic depolarizations at the synapses they pass (Hoffman et al., 1997; Magee and Johnston, 1997; Hebb, 2005; Dan and Poo, 2006; Feldman, 2012). The back-propagating action potential (bAP), therefore, satisfies the post-signaling condition of STDP (Levy and Steward, 1983). The learning signals of bAPs resemble backpropagation through time (BPTT) with surrogate gradients and target-prop algorithms (Werbos, 1990; Sacramento et al., 2018; Neftci et al., 2019; Lillicrap et al., 2020). Learning in dendritic regions distant from the soma is problematic because bAP does not reach the most distal dendrites or reaches them only in an attenuated manner. Therefore, a bAP is unlikely to provide the necessary depolarization to contribute to the induction of LTP at the most distal synapses (Krueppel et al., 2011). Plasticity can also be triggered by depolarizations originating from sources other than bAPs in the postsynaptic neuron, e.g., dendritic Ca_2+_ spikes (Golding et al., 2002; Kampa et al., 2006; Letzkus et al., 2006), N-methyl-D-aspartate (NMDA) spikes (Gordon et al., 2006; Brandalise et al., 2016), or excitatory postsynaptic potentials (EPSPs) alone for LTP induction at the most distal synapses (Golding et al., 2002; Lin et al., 2008; Sjöström et al., 2008; Weber et al., 2016; Kim et al., 2018). Although many biophysical details of excitatory synaptic long-term plasticity remain to be fully elucidated, it is generally accepted that postsynaptic Ca_2+_ pulses play a fundamental role. A possible plasticity mechanism, called backpropagation-activated Ca_2+_ (BAC) firing, involves coincidence of strong proximal and distal inputs that may lead to dendritic spikes and bursts of axosomatic APs (Larkum et al., 1999; Hamilton et al., 2010). Synapses that cooperate on their quest to associate different inputs potentiate, whereas synapses that do not cooperate and/or do not succeed to establish an associational signal depress. BAC firing potentiates those synapses that cause it, thereby increasing the probability that this selected subset of synapses leads to BAC firing at the next time they are active (Larkum, 2013). Calcium-dependent dendritic spikes with attenuating amplitude (dCaAPs) and dendritic Na_+_ spikes allow NMDAR dependent LTP in distal synapses (Gidon et al., 2020). Ca_2+_ pulses of short duration and high amplitude induce LTP (Evans and Blackwell, 2015). Low levels of Ca_2+_ lead to no changes in synaptic strength, medium levels cause LTD, and high levels lead to LTP (Lisman, 1989; Artola et al., 1990; Artola and Singer, 1993; Shouval et al., 2002; Graupner and Brunel, 2012). Potentiation can occur when an NMDA spike (also called a dendritic plateau potential) is generated. These plateau potentials provide a long and sufficiently high depolarization that leads to potentiation without generating postsynaptic action potentials. Plateau potentials can control plasticity at other synapses. Because they are more readily evoked in the terminal regions of basal dendrites, they undergo considerable attenuation and cause only subthreshold events at the soma. This long subthreshold plateau reduces the depolarization required to reach the spike threshold and, therefore, allows other weak inputs to reach the threshold. Active conductance in dendrites can normalize the efficacy of distal synapses and democratize dendrites by making distal and proximal synapses equally efficient in influencing somatic firing. Strong dendritic tapering attenuates electrotonic attenuation to such an extent that the dendritic tree becomes approximately isopotential (Otopalik et al., 2019). Therefore, dendritic structures may avoid attenuation. Inhibitory synapses on the dendritic tree prevent backward propagation of APs, while forward propagation remains intact (Wilmes et al., 2016; Iascone et al., 2020). Once a dendritic spike is generated, local inhibition can terminate it either transiently or permanently, depending on their relative timing. The classical “pre-/post-spike-timing-dependent-plasticity” (STDP) rule states that plasticity depends on pre-synaptic activity before and a sensed postsynaptic potential afterwards (Yang and Dani, 2014). STDP requires the generation of somatic Na_+_ spikes and their backpropagation into the dendrites. Pair-based STDP cannot account for activity-dependent learning with weak inputs, which are not powerful enough to evoke bAPs. Finally, an increasing number of experimental studies have revealed plasticity mechanisms that do not rely on bAP but instead on local postsynaptic dendritic spikes or sub-threshold events for dendritic spikes (Ebner et al., 2019). Plasticity of distal feedback-associated synapses is a hot topic in studies exploring the idea of deep learning in the brain (Guerguiev et al., 2017; Richards et al., 2019).

## 3. Methods

**Figure 2:**
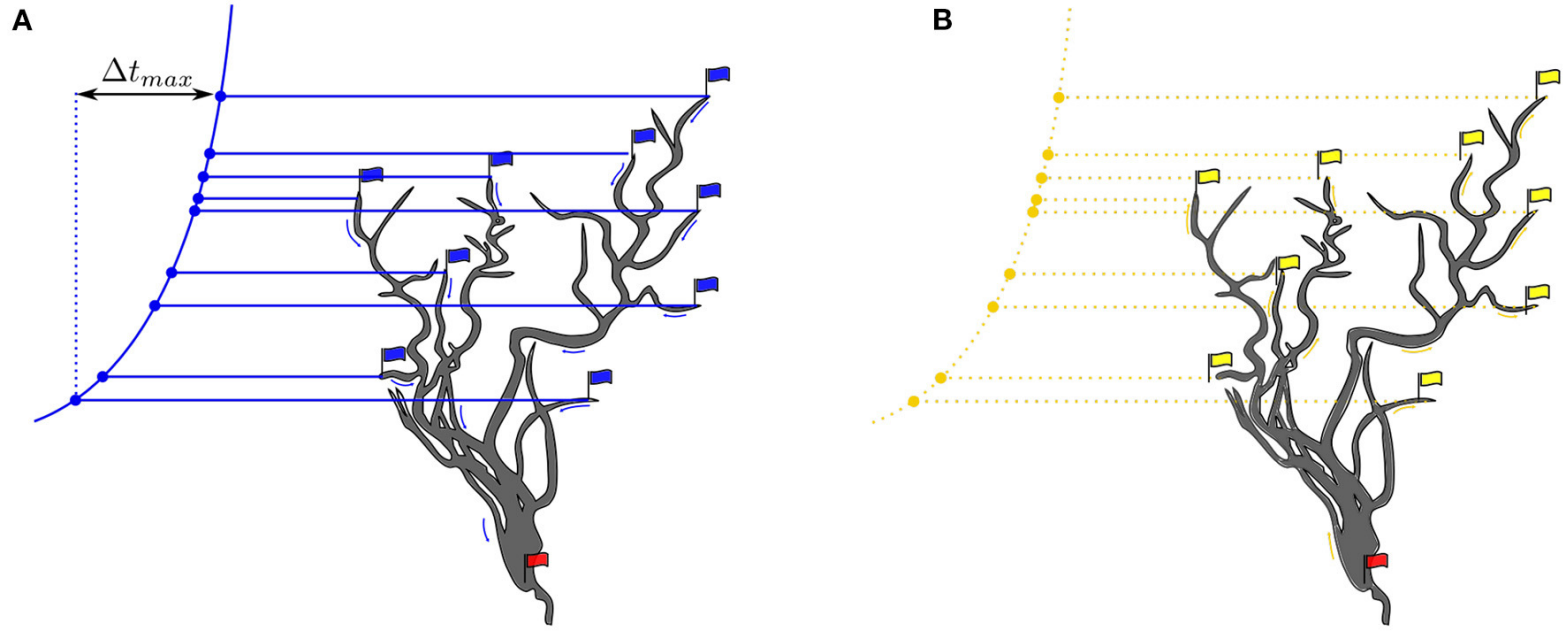
**(A)** Blue flags indicate race starts, beginning with FA and ending with LA. The red flag is triggered by an action potential event. The variation principle is given by the formula given in Equation 1. **(B)** The red flag heralds the start of a backpropagation signal that propagates to the most distal dendrites. Yellow arrows represent dendritic signal propagation velocities. Yellow flags indicate synapses in active sensitized state.

The octopus' cell is represented by a soma with dendritic branches and modeled using state-dependent temporal logic operators. The soma functions like a sample-and-hold flow meter with a blocking mode that opens the valve when the correct coincidence condition is met. The ANF axons are considered conductive long leads and the auditory nerve bundle is considered a ribbon cable with spliced ends. Conducting axons initiate synaptic anchoring of neuroligins, which adhere to the dendritic spines on the axons like clothespins, as the first step to initiate synaptogenesis. The number of potential synapses can be estimated from the approximate site of contact of an ANF with a dendrite that are close enough to each other and formulated by an action cross-section parameter. The synchrony transfer function of the octopus' cell as a function dependent of the ANF volleys will be computed in the time domain. The octopus' cell's model is represented by stateful temporal logic operators executing dendritic signal fluxes that obey Kirchhoff's bifurcation laws, whose sum potentials bounce off the soma and induce synaptic potentiation through pre-post(-post) spike cascades. This model replaces the one described in Harczos and Klefenz (2018). The core behind stateful temporal logic is the encoding of information in the timing of events and their voltage level amplitudes. The operators that form the set of stateful temporal logic are **Min**(FirstArrival), **Max** (LastArrival), Constant Delay (**D**), Inhibit (**I**), Reset (**R**), and Coincidence (**C**) (Smith, 2018). For state-based temporal logic, the operator Memory (**ON/OFF**) is added (Madhavan and Stiles, 2020; Madhavan et al., 2021). A system **S** goes through a sequence of states in time (Tzimpragos et al., 2020) controlled by the named operators. Since we consider the octopus' cell model as discrete in time, we model the cell as a system **S** and show that five elementary operators are sufficient to establish its functional behavior. Inhibit is unused since the model can be formulated without it without tradeoffs. Reset is also absent, since a fixed extinction time is assumed after signal onset at a synapse. The extinction time for returning to the **OFF** state is set to 2 ms after synaptic excitation. This corresponds to the maximum return time of 2 ms. The constant delay is derived from the Poisson firing statistics of octopus cells and was determined in Aubie's modified model as 18 μs standard deviation (SDEV) (Klefenz and Harczos, 2020). The dendritic path lengths are expressed as n delays (**D**) with a uniform delay constant **D** of 18 μs. We can make the following assumptions for the operators and constants:

- **Min** condition is given by the first arrival (FA) of an ANF spike at a synapse.
- **Max** condition is given by the last arrival (LA) of an ANF spike at a synapse (Figure 2).
- t(FA − LA) is the causal time window in which collective interactions can occur.
- t = (FA − LA) and the uniform jump size of 18 microseconds determine the number of discretized time steps.
- t = (FA − LA) is approximately 4 milliseconds for a local receptive field in the low frequencies, resulting in a simulation run of 222 time steps for the forward run.

Coincidence (**C**) is satisfied when tokens travel through the dendritic conductors and cross a predefinable activation threshold in a fixed time window.

At token start, a blue start flag is raised. The race end flag is hoisted at the moment when the threshold has been crossed indicated by setting the red flag at that moment in Figure 2.

Memory is assigned to the synapses formed between ANFs and octopus dendrites. A synapse is in its active sensitized state, if evoked by a signal flow from its associated ANF. This active sensitized state of a synapse is described as **ON**, else **OFF**. The intermediate synaptic weight and its final convergence to high (1) or low (0) is learned by an STDP rule.

### 3.1. Dendritic Calculation

A recent renaissance of dendritic computation has emerged through proposals of new model variants (Ostapoff et al., 1994; Voelker and Eliasmith, 2018; Payeur et al., 2019; Wybo et al., 2019; Gidon et al., 2020; Lepicard and Ann Piskorowski, 2020; Moldwin and Segev, 2020; Poirazi and Papoutsi, 2020; Takahashi et al., 2020; Banerjee et al., 2021; Callan et al., 2021; Martin et al., 2021; Moldwin et al., 2021; Stöckel and Eliasmith, 2021; Yang et al., 2021b). The octopus' cell acts as a synchronizer (McGinley et al., 2012). Functional simplicity is the overarching goal in constructing the dendritic tree with state-dependent temporal logic operators to sublimate many subtle details of neural morphology and rate kinetic dynamics. The tree is constructed simply by concatenating unit-base elements into unbalanced variable-length branches. The innervation patterns of the ANFs are given by the input activity matrix A(*S*_*ij*_(*t*)). From the activated synapses, Dirac-like subthreshold voltage signals propagate down the dendrites to the soma, can collectively generate APs, and can produce their own unsupervised teaching signal by backpropagation into the dendrites. Kirchhoff's rules apply at the dendritic junctions, and attenuation and scattering of the signals are not considered. The current flows converge at the junctions and swell after the junctions. The soma is depolarized when the charge currents from the dendritic tree arrive synchronously and enough charge is accumulated to overcome the depolarization threshold. Backpropagating action potentials velocities are in the range of 226 μm/ms, which is 0.2–0.3 μm/μs (Senzai and Buzsáki, 2017) and 508 μm/ms for apical dendrites of layer five pyramidal neurons (basal dendrites, 341 μm/ms) (Nevian et al., 2007) and is similar to or lower than the estimated velocity in apical dendrites of other hippocampal principal neurons (Spruston et al., 1995b; Kim et al., 2012).

### 3.2. Variation Principle of Synchronization

The condition that a delay trajectory in the receptive field of an octopus' cell leads to the same arrival time t_*arrival*_ at the soma for a vesicle in a dendrite branch *i* is given by:


(1) $$\begin{array}{r} {\text{t}}_{\text{arrival}}={\text{t}}_{i}+\Delta {\text{t}}_{i} \end{array}$$

Where t_*i*_ is the arrival time at the synapse connected to that branch and Δt_*i*_ the time the spike travels along the branch to the soma. Earlier arrival times are compensated by longer travel times in the partially shared dendrite pathways (Figure 2). The variation principle is to adjust all path lengths and velocities so that the formula is satisfied for each synapse, or at least for a subset of synapses.

### 3.3. Backpropagation

The collectively triggered action potential travels along the axon and, simultaneously, a potential propagates backward from the soma into the dendrites until the most distant synapses are reached (Figure 2) (Brunner and Szabadics, 2016). The same paths defined by the delay elements (**D**s) tree are taken on the backpropagation path. Attenuation and dispersion of the backpropagation signal flow are neglected in this work.

### 3.4. Synaptic Learning Rule

Learning at a synapse is given by a causal associative pre-post spike-timing dependent plasticity rule (STDP). A synapse is activated by presynaptic glutamatergic vesicles from the presynaptic ANF and enters an active sensitized state for a while (preconditioning). A triggered action potential at the soma generates a backward propagating signal into the dendrites (postconditioning signal) (Figure 2). The (pre, post) condition is satisfied when the synapse is in its active sensitized state and the backpropagating signal occurs before desensitization (Figure 2).

The weight update rule simplifies to:


(2) $$\partial w_{j}=\varepsilon \nu_{\text{Active}}\;\tau_{\text{backprop}}$$

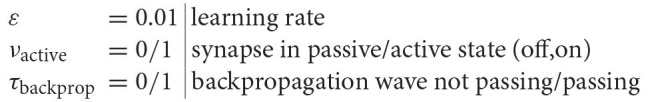


Synapses consist of both AMPA and NMDA channels. The number of AMPARs is set to zero at the beginning. The maximum AMPAR conductance is chosen to be equal to the maximum NMDAR conductance. The weights of active synapses are updated when a somatic bAP is triggered. The weights of the synapses converge to a state of maximum conductance 1 or rest at the minimum state 0. The learning rate is adjustable and chosen such that one hundred backpropagation signals drive the synaptic weight *w*_*j*_ to its maximum 1.

### 3.5. Functional Implementation in a Numerical Model

To validate the proposed learning rule, we created a numerical model in the Python programming language (Python 3). The focus is on realizing a discrete-time model in an event-driven environment. The activation inputs of the octopus cells are given by the matrix A(*S*_*ij*_(*t*)).

**Figure 3:**
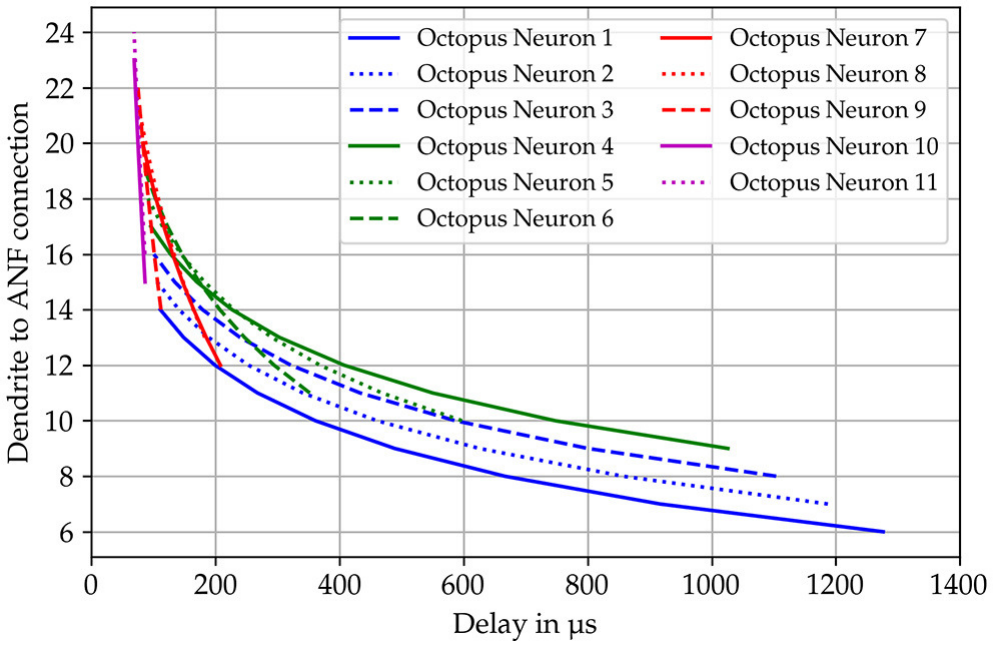
Dendritic delays are calculated according to the formula published by Greenwood (1990). The specific delay is shown on the x-axis and the connected auditory nerve fiber on the *y*-axis. The templates for the delay curves are superimposed as in the actual model.

Stuart et al. (2016) give a detailed description of the morphology and functionality of dendrites in their compendium. According to this, the dendritic tree consists of directed graph elements (Mel et al., 2016) that are combined to form a more complex network. The soma is the root and the graph divides to reach all synaptic sites. Each node knows its child and predecessor nodes to realize conductive forward and backward propagation paths from synapses to the soma. The dendrites form a directed acyclic graph that is directed to either the soma or synaptic connections depending on the propagation state (forward/backward) (Figure 2). The dendritic tree is simplified to individual linearized dendrites in the form of tapped delay lines with a static delay of 18 μs per simulation time step. The dendritic delays are chosen to follow a logarithmic curvature given by the Greenwood formula (Greenwood, 1990). The delay values are assigned to each octopus' dendrites in a way that the octopus delays overlap over the receptive field (Figure 3).

The tokens of a trajectory travel to the soma, which accumulates all inputs over a short time window and generates an action potential when a certain threshold is crossed. When this event occurs, a wave is backpropagated, and each time the weight values of all activated synapses are increased by the value of the learning rate. The repeated backpropagation waves bring the synaptic weights to their saturation value of 1.0, putting the system in a steady state. The soma leakage is modeled by decreasing the membrane potential of the soma by a fixed decay value at each time step. The decay must be chosen very carefully because too large a value will cause the soma to see more than one trajectory and produce spikes at the output with a multiple of the central time interval, and too small a value would prevent the soma from firing at all.

**Figure 4:**
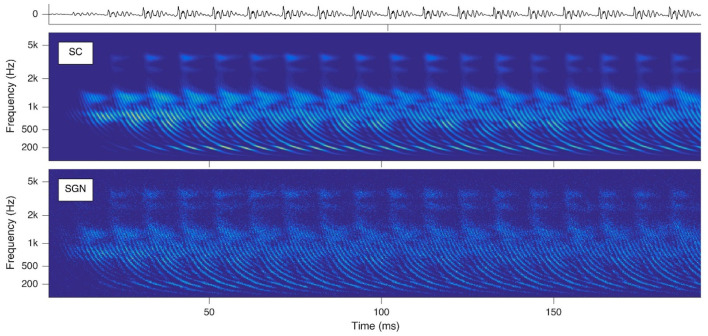
Cochleagrams with quasi-stationary repeating patterns for a short section of the vowel “a” sung by a male singer on the note G2. Top: waveform of the audio signal. Middle: Probability (ascending from blue to green to yellow) of neurotransmitter release into the synaptic cleft (SC) as a function of time and location within the cochlea. Bottom: Action potentials of spiral ganglion (SGN) neurons. Note that the ordinate shows the characteristic frequency of the basilar membrane model at the corresponding cochlear location [reprinted from Harczos and Klefenz (2018)].

To provide biologically plausible input for out numerical model an auditory front-end is used called SAM. SAM stands for Stimulation based on auditory modeling and it creates cochleagrams from a given prerecorded sound file as shown in Figure 4 for the vowel “a” sung by a woman on the note G2.

Our program package is designed for maximum flexibility to quickly create and study different topologies for pitch perception.

## 4. Proof of Principle

**Figure 5:**
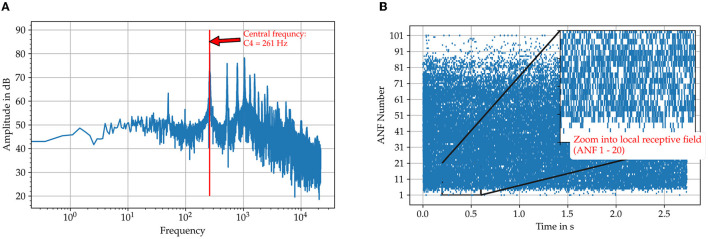
Illustration of the input data for the sung vowel “a” with a pitch C4 from the Fraunhofer dataset. **(A)** The FFT spectrum calculated with the FFT algorithm included in the Python package SciPy. **(B)** The spike trains on the auditory nerve fibers originating from the SAM model. Those are used as direct input to the octopus' neuron model.

The model and our hypothesis is tested with two audio samples taken from the Fraunhofer dataset also used in Klefenz and Harczos (2020) for terms of comparability. The first one is a recording of a female singer, who sings the vowel “a” with a pitch of C4 (261Hz) and the second a pure sine tone with the same pitch. The time interval for the center frequency of the samples is about 3.83 ms. Figure 5 shows the cochleagram for the first sample and the spectrum. The energy is not concentrated solely at the pitch frequency (Figure 5) but also at its harmonics. This makes it difficult for the neuron to detect the interval of the base tone from the superimposed spike intervals accumulated in the spike pattern of auditory nerve fibers.

**Figure 6:**
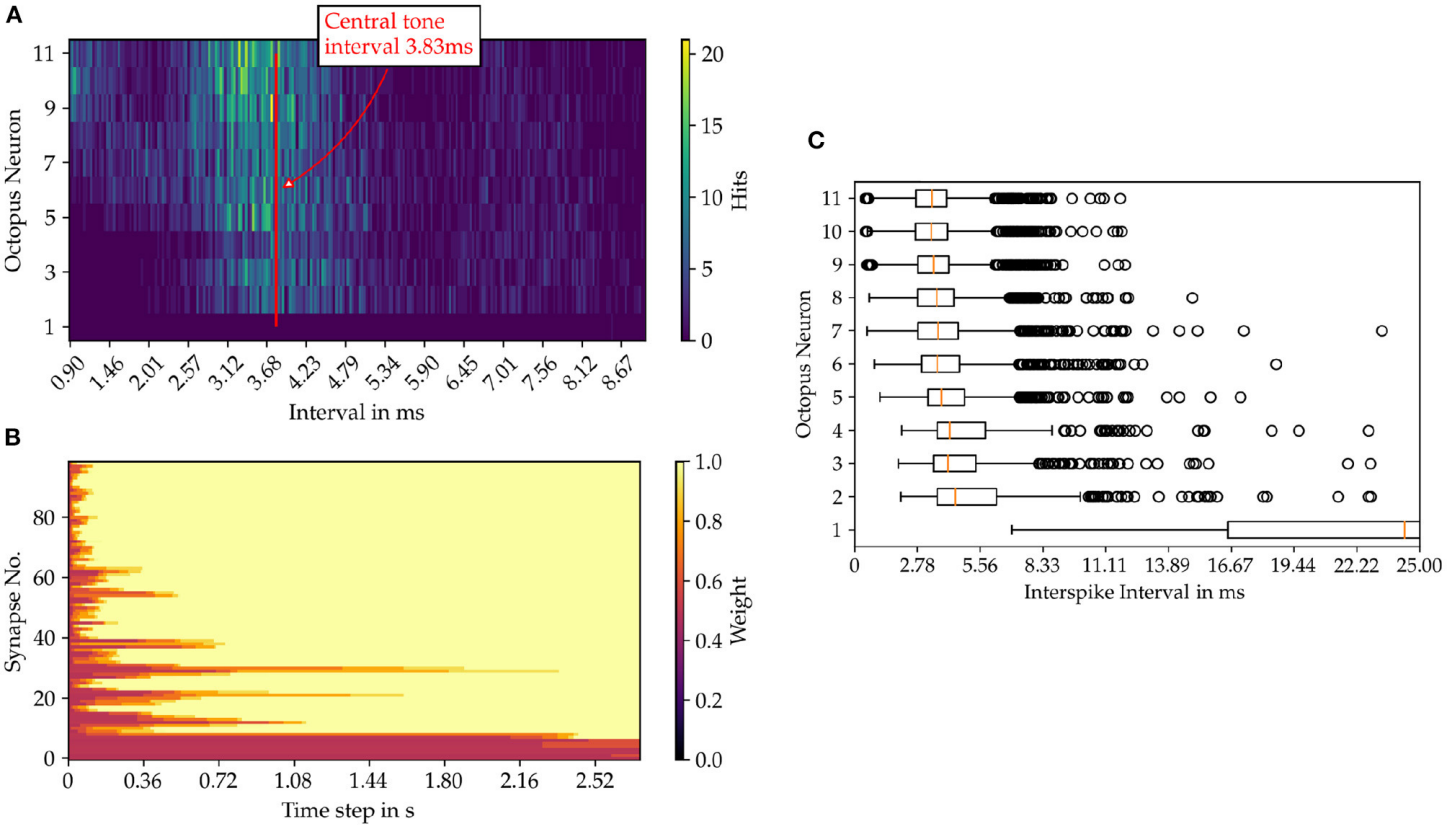
Results from the model simulation of the sung vowel “a” sample in pitch C4. **(A)** Inter spike intervals (ISI) of the octopus neuron layer. In image **(B)** the time evolution of the synaptic weights are shown and in **(C)** the statistics of the ISIs of the octopus layer is depicted.

**Figure 7:**
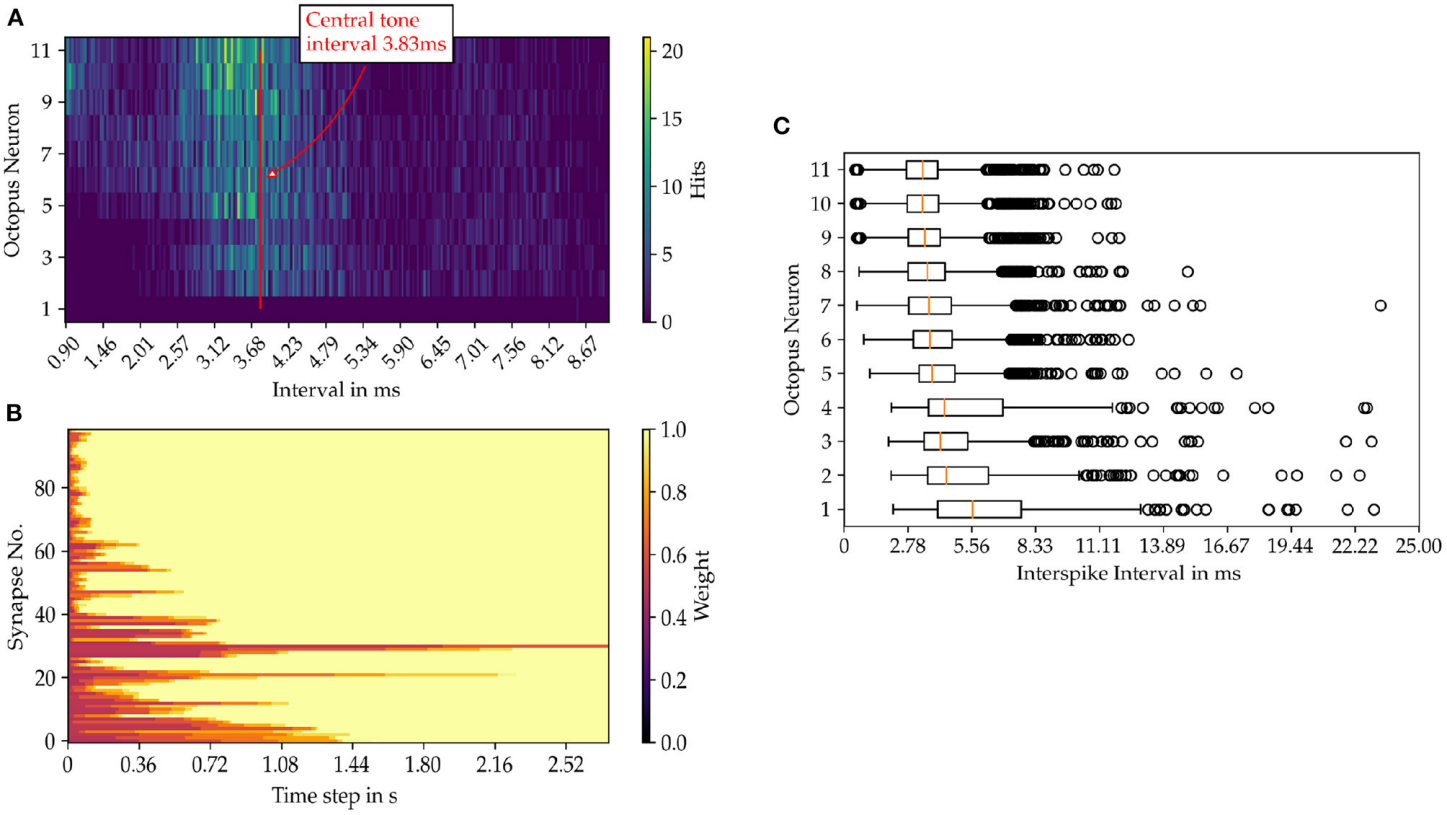
Results from the model simulation of a pure sine tone with the pitch C4. **(A)** Inter spike intervals of the octopus layer. In image **(B)** the time evolution of the synaptic weights are shown and in **(C)** the statistics of the ISIs of the octopus layer is depicted.

For each octopus' neuron the histogram of interspike intervals is constructed as shown in Figure 6 for the sung vowel a with a pitch of C4 and in Figure for a pure sine tone with the same pitch (Figure 7). Summing vertically over the partial histogram entries of all octopus cells, the global histogram maximum is reached at 3.83 ms and the distribution concentrates around the central time interval.

### 4.1. Learning Inter-Spike Interval Histograms

In the auditory model, 101 ANFs are arranged tonotopically along the frequency axis from the lowest to the highest frequency (Harczos, 2015). The spectral interval from C3 to G5 is examined using the presented model (Harczos and Klefenz, 2018). There are eleven octopus cells in this interval, each connected to nine separate ANFs. This wiring scheme represents a local receptive field for each octopus' cell. Adjacent receptive fields partially overlap and share multiple ANFs as inputs (Klefenz and Harczos, 2020).

**Figure 8:**
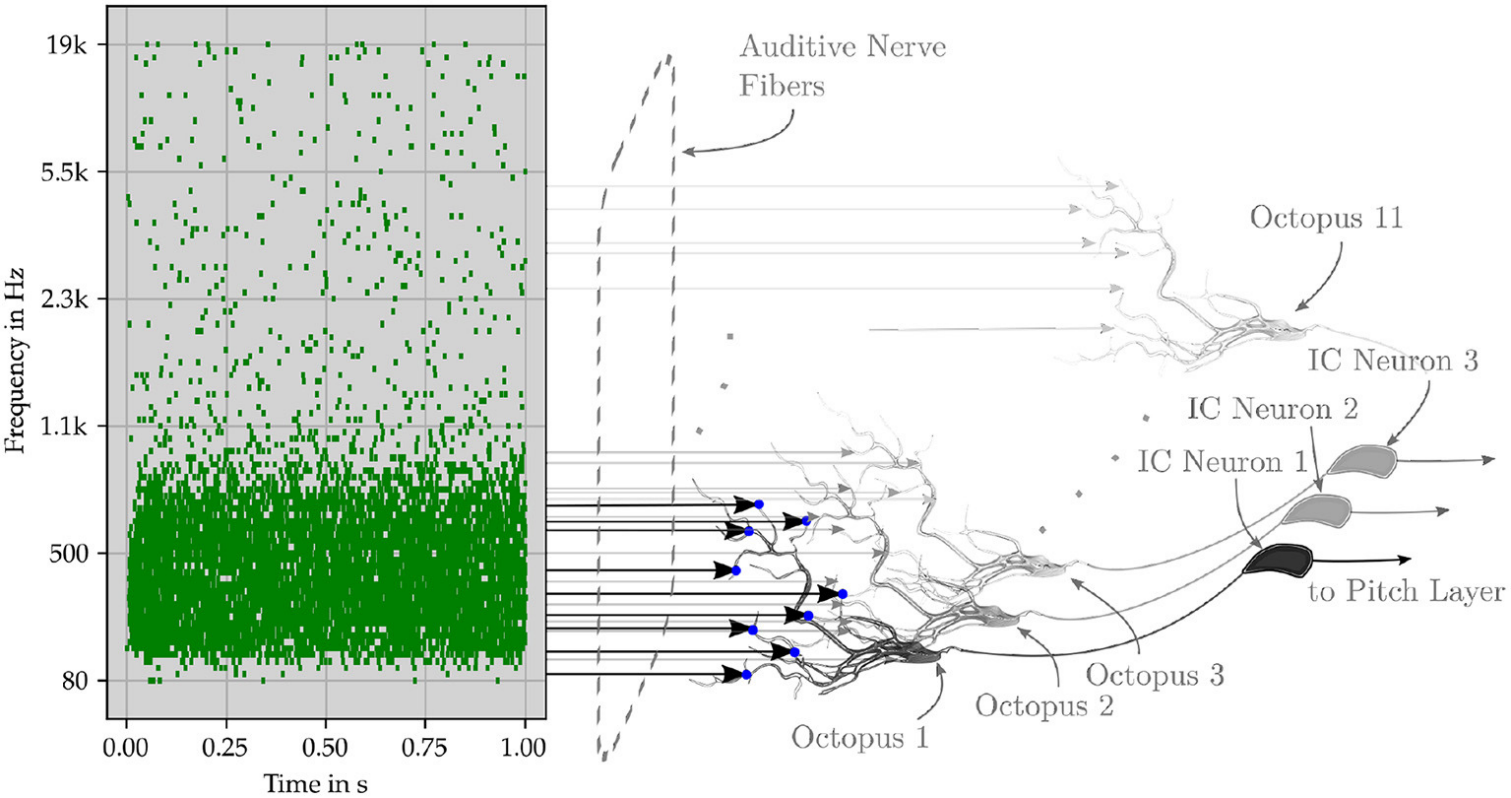
Schematic representation of the perceptual network. The original pitch is encoded by successive trajectories in the ANF. The event signals are collected in an octopus neuron layer, in which the neurons have overlapping synaptic connections to the fiber. After the octopus layer, the interspike interval of the neuron output is centered around the central frequency, while the interspike interval of the input has several maxima. The following layer of inferior colliculus (IC) neurons is triggered by specific spike intervals. Pitch neurons perceive pitches from the collective spikes of the IC neurons.

An octopus' cell fires when a delay template segment matches a global trajectory and the corresponding synaptic connections are strengthened (Shamma and Dutta, 2019). Multiple octopus cells fire a series of spikes together when exposed to a common global trajectory and local receptive conditions are met. For quasi-stationary acoustic signals, nearly the same ANF trajectories occur repeatedly, and ANF firing patterns are nearly identical, as are the firing patterns of octopus cells. Two successive trajectories trigger firings of octopus cells at two different times in succession, forming inter-spike intervals (ISI). The entire process sequence is shown in a schematic diagram as an example in Figure 8.

**Figure 9:**
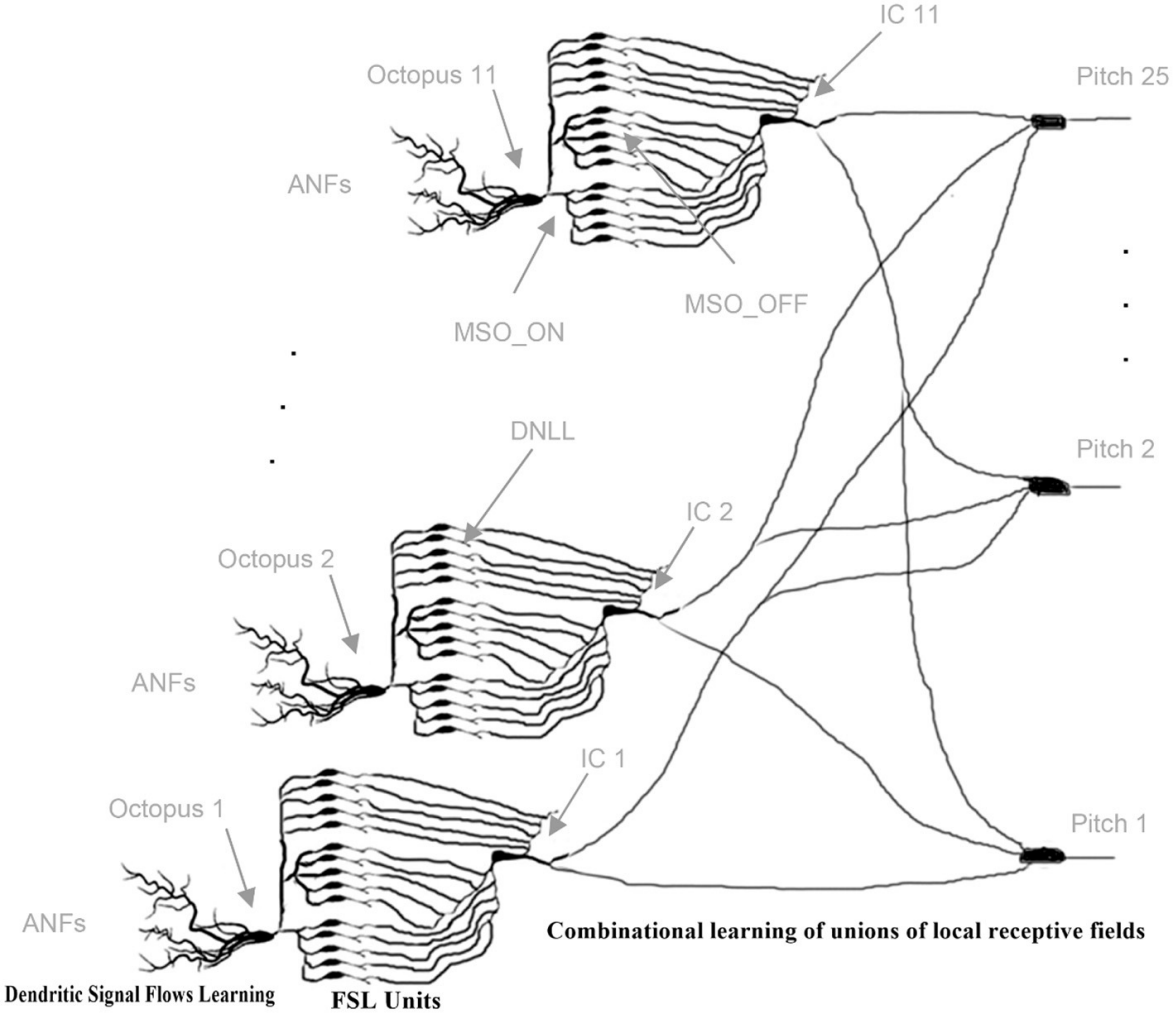
Cascaded, layered learning: initially, dendritic signaling fluxes are learned individually and octopus cells begin to spike; gradually, combinatorial associations of ITNs are learned. FSL units are hardwired [Adapted reprint from Klefenz and Harczos (2020)].

Their time intervals are converted into a latency code for the first time by the transmission circuits between the octopus' cell and the ITN (Figure 9). The ITN fires first at the shortest time interval and last at the longest interval. Each intervening time interval is indicated by the corresponding FSL time. The collective task is to create, store, and interpret ISI histograms (Harpaz et al., 2021). The pitch neurons are connected to the corresponding bins. Each pitch neuron collects and counts the entries in its associated histogram bin. Inter-spike intervals are linearly proportional to FSL times. A start condition flag starts a clock counter at the FSL time of the corresponding minimum inter-spike interval (Verzi et al., 2018). Each pitch neuron fires when the entries in its FSL bins are above a fixed threshold number. The involved innervating synapses from ITNs to pitch neurons are increased by *w*_*ij*_ during the learning phase for this event. Collective learning of pitch by observing and interpreting associations of ICs by pitch neurons has been extensively studied in Klefenz and Harczos (2020).

## 5. Discussion

The programmed model is able to determine the central time interval, exemplified by a sung vowel of “a” on the tone C4. The histogram of the interspike intervals for each octopus' neuron certainly hits the central interval of 3.83 ms, but the intervals scatter quite strongly. This can be optimized by more careful choice of delay templates for the dendritic branches. The resulting intervals are detected by tuned timer neurons that respond to a specific time interval and generate a spike when an interval with a certain uncertainty is hit. A group of firing interval-tuned neurons indicates a detected pitch and leads to detection by the pitch neurons. This is not part of this work and will be presented in future work. However, the results indicate, that the octopus neurons are able to improve the pitch detection for subsequent layers by concentrating the spike rate at the central interval.

The stateful temporal logic algebra system is realizable as a neuromorphic circuit built with the seven building blocks **FA**, **LA**, **D**, **C**, **M**, **I**, **R** and is implementable for various hardware target architectures. It is especially suited for implementation in CMOS (Nair et al., 2020; Han et al., 2021), FPGA (Yang et al., 2021a), and quantum-based hardware (Varadarajan, 2014; Gonzalez-Raya et al., 2019; Hamilton et al., 2019; Shi et al., 2019; Lamata, 2020; Marković et al., 2020) as nanobridge atomic switch FPGAs (Demis et al., 2015; Sharma et al., 2021) superconducting accelerators (Tzimpragos et al., 2020; Vakili et al., 2020; Feldhoff and Toepfer, 2021), superconducting nanowires (Toomey et al., 2019), nanowire networks (Diaz-Alvarez et al., 2020; Kendall et al., 2020; Kuncic et al., 2020; Li et al., 2020; Milano et al., 2020; Dunham et al., 2021; Kendall, 2021) and memristors (Sanz et al., 2018; Woźniak et al., 2020).

## 6. Conclusions

The octopus' cell model can be described with state-dependent temporal logic operators and simulated numerically. An octopus' cell model in a local receptive field adapts to a given trajectory by STDP learning with synaptic pre-activation and the dendritic return signal as post-condition. There is no need to distinguish between learning and retrieval phases. Pitch learning occurs in a cascade fashion: first, octopus cells respond individually by self-adapting to specific trajectories in their local receptive fields, then associations of octopus cells are collectively trained to discriminate pitch.

## Data Availability Statement

The original contributions presented in the study are included in the article/supplementary material, further inquiries can be directed to the corresponding author/s.

## Author Contributions

FK had the idea and wrote the main part of the manuscript. FF programmed the model, created the plots and contributed in writing and proofreading of the manuscript, and during the work. TH provided the SAM model for input data generation and also proofread the manuscript. HT contributed some ideas and proofread the manuscript. All authors contributed to the article and approved the submitted version.

## Funding

This work was partially supported by the Carl Zeiss Stiftung in the framework of the project Memwerk and the project Quantum Hub Thuringia, grant number 2021 FGI 0048.

## Conflict of Interest

TH was employed by audifon GmbH & Co. KG. The remaining authors declare that the research was conducted in the absence of any commercial or financial relationships that could be construed as a potential conflict of interest.

## Publisher's Note

All claims expressed in this article are solely those of the authors and do not necessarily represent those of their affiliated organizations, or those of the publisher, the editors and the reviewers. Any product that may be evaluated in this article, or claim that may be made by its manufacturer, is not guaranteed or endorsed by the publisher.

## Footnotes

^1^N-methyl-D-aspartate (NMDA) receptors, α-amino-3-hydroxy-5-methyl-4-isoxazolepropionic acid receptors (AMPAR) and gamma-Aminobutyric acid (GABA).
